# Lack of correlation between the optimal glycaemic control and coronary micro vascular dysfunction in patients with diabetes mellitus: a cross sectional study

**DOI:** 10.1186/s12933-015-0269-1

**Published:** 2015-08-14

**Authors:** Luis Felipe Valenzuela-Garcia, Yasushi Matsuzawa, Jaskanwal D S Sara, Taek-Geun Kwon, Ryan J Lennon, Lilach O Lerman, Rafael J Ruiz-Salmeron, Amir Lerman

**Affiliations:** Heart Center, Virgen Macarena Hospital, Avenida Doctor Fedriani, nº 3, 41007 Seville, Spain; Division of Cardiovascular Diseases and Department of Internal Medicine, Mayo College of Medicine, 200 First Street SW, Rochester, MN 55905 USA; Division of Biomedical Statistics and Informatics, Mayo College of Medicine, 200 First Street SW, Rochester, MN USA; Division of Nephrology and Hypertension, Mayo Clinic, 200 First Street SW, Rochester, MN USA

**Keywords:** Endothelial dysfunction, Diabetes mellitus, Coronary microcirculation

## Abstract

**Background:**

Coronary microvascular dysfunction (CMD) is associated with cardiovascular events in type 2 diabetes mellitus (T2DM). Optimal glycaemic control does not always preclude future events. We sought to assess the effect of the current target of HBA1c level on the coronary microcirculatory function and identify predictive factors for CMD in T2DM patients.

**Methods:**

We studied 100 patients with T2DM and 214 patients without T2DM. All of them with a history of chest pain, non-obstructive angiograms and a direct assessment of coronary blood flow increase in response to adenosine and acetylcholine coronary infusion, for evaluation of endothelial independent and dependent CMD. Patients with T2DM were categorized as having optimal (HbA1c < 7 %) vs. suboptimal (HbA1c ≥ 7 %) glycaemic control at the time of catheterization.

**Results:**

Baseline characteristics and coronary endothelial function parameters differed significantly between T2DM patients and control group. The prevalence of endothelial independent CMD (29.8 vs. 39.6 %, *p* = 0.40) and dependent CMD (61.7 vs. 62.2 %, *p* = 1.00) were similar in patients with optimal vs. suboptimal glycaemic control. Age (OR 1.10; CI 95 % 1.04–1.18; *p* < 0.001) and female gender (OR 3.87; CI 95 % 1.45–11.4; *p* < 0.01) were significantly associated with endothelial independent CMD whereas glomerular filtrate (OR 0.97; CI 95 % 0.95–0.99; *p* < 0.05) was significantly associated with endothelial dependent CMD. The optimal glycaemic control was not associated with endothelial independent (OR 0.60, CI 95 % 0.23–1.46; *p* 0.26) or dependent CMD (OR 0.99, CI 95 % 0.43–2.24; *p* = 0.98).

**Conclusions:**

The current target of HBA1c level does not predict a better coronary microcirculatory function in T2DM patients. The appropriate strategy for prevention of CMD in T2DM patients remains to be addressed.

## Background

Cardiovascular disease is a major cause of morbidity and mortality in patients with type 2 diabetes mellitus (T2DM) [1]. No clinical trial has provided conclusive evidence that improved glycaemic control reduces the risk of adverse events [2] and recent studies have even shown potential risks associated with intensive glycaemic control [3].

Coronary micro vascular dysfunction (CMD) is associated with cardiovascular related morbidity and mortality in T2DM patients [4]. Endothelial dysfunction (ED) plays an important role in the progression of atherosclerosis in DM patients [5]. Several trials have demonstrated that intensive glycaemic control reduces the progression of micro vascular disease including manifestation and progression of nephropathy, end stage renal disease and retinopathy [6]. However, the benefit of achieving an optimal haemoglobin A1c (HbA1c) level to minimize or reverse the manifestation and progression of CMD in T2DM patients has not been reported. Previous evidence on the subject is scarce and limited [7–9]. In the present study we aimed to evaluate the effect of achieving the current target of HbA1c < 7 % on the prevalence of CMD in a population of T2DM patients with chest pain and non-obstructive CAD. We also analysed other clinical factors in addition to lowering HbA1c potentially associated with CMD in these patients.

## Methods

This retrospective study was approved by the Mayo Foundation Institutional Review Board. In keeping with Minnesota statute, only patients who granted use of their records for research purposes were included. Between January 1st 1993 and June 30st 2013, a total of 1,579 patients referred for assessment of chest pain suspected of cardiac ischemia underwent coronary angiography and invasive endothelial function testing at Mayo Clinic. Most patients underwent a non-invasive test for detection of ischemia, however a positive result was not an inclusion criteria in our study since a negative non-invasive stress test does not rule out coronary vasomotor dysfunction in symptomatic patients with nonobstructive coronary artery disease [10]. Patients with the following were excluded: greater than 40 % diameter stenosis of any coronary artery; acute coronary syndrome; uncontrolled hypertension; left ventricular ejection fraction of 50 % or less; left ventricular hypertrophy and severe endocrine, hepatic, renal or inflammatory disease. For the purpose of this study we excluded 476 patients because of missing values of HbA1c at the time of cardiac catheterization, and 36 patients who had not measurements available for both coronary blood flow (CBF) and coronary flow reserve (CFR). From the remaining 1,067 patients, we analyzed those with diagnosis of T2DM (n = 100) who were categorized according to HbA1c level in those having an optimal (HbA1c < 7 %) vs. suboptimal (HbA1c ≥ 7 %) glycaemic control as evaluated with a single measurement of this parameter at the time of catheterization. As a comparison group (n = 214) we considered those not matching diagnostic criteria for type 1 diabetes mellitus [11], Metabolic Syndrome, [12] or Pre-Diabetes, [13]. The duration of Diabetes was specifically addressed through a detailed revision of previous clinical records [14].

Patients presented to the cardiac catheterization laboratory in the fasting state and all cardiovascular medications, including nitrates and calcium channel blockers, were discontinued for at least 48 h. At the time of catheterization, blood samples were drawn to measure the biochemical profile and biomarkers including Lipoprotein A, high sensitivity C Reactive Protein and Homocysteine. Informed consent was obtained from each patient and the study protocol conformed to the ethical guidelines of the 1975 Declaration of Helsinki as reflected in a priori approval by the institution’s human research committee.

Routine diagnostic coronary angiography was performed in all patients using standard clinical protocols and reviewed prior to the infusion of any pharmacological agents. In cases where the severity of stenosis was uncertain, quantitative coronary angiography was used. All patients underwent evaluation of endothelial-dependent CBF and endothelial-independent CFR as previously described [15]. Following intravenous infusion of 5,000–7,000 U of heparin, a Doppler guidewire (Flo-wire, Volcano) 0.014 inches in diameter within a 3-F. Slip-Cath Infusion Catheter (Cook Medical) was positioned into the mid-portion of the left anterior descending coronary artery, 2–3 mm distal to the tip of the infusion catheter. Heart rate and mean arterial blood pressure were continuously monitored throughout each procedure. Baseline peak velocity was recorded using intravascular Doppler assessment after which intracoronary bolus injections of increasing doses (18–72 µg) of adenosine was administered through the guide catheter until maximal hyperemia was achieved. The maximal mean peak velocity was then recorded and CFR ratio calculated by dividing the mean peak velocity following the administration of adenosine by the baseline mean peak velocity.

After a 5-min equilibration period, acetylcholine was infused at concentrations of 10^−6^, 10^−5^ and 10^−4^ M (to achieve estimated coronary bed concentrations of 10^−8^, 10^−7^ and 10^−6^ M respectively) for 3 min at each concentration to assess endothelium-dependent increase in CBF. Infusions were performed using a Medfusion^®^ 3500 pump to maintain infusion rates of less than 1 % of the estimated CBF. Doppler measurements of mean peak velocity were performed after each infusion followed by repeat coronary angiography. Coronary artery diameter was measured by an independent investigator blinded to Doppler velocity data and CBF was then calculated using the following, as previously described: CBF = (mean peak velocity/2)(coronary artery diameter/2)^2^. Maximal CBF following the infusion of acetylcholine was then divided by the CBF at baseline to give the change in CBF as a ratio. Impaired endothelial-independent micro vascular function was defined as a CFR ratio in response to adenosine of 2.5 or less Impaired endothelial-dependent micro vascular function was defined as the maximal increase in CBF following infusion of any dose of acetylcholine compared to baseline of 50 % or less, giving a ratio of 1.5 or less.

For quality control, all angles, skew rotation and table height were kept constant during each procedure. Furthermore, the distance between the image intensifier and x-ray tube relative to the patient was kept constant. Measurements were performed in the segment 5 mm distal to the tip of the Doppler wire and following each infusion, the diameter was measured in the same segment of the vessel [15].

### Statistical analysis

Continuous variables are presented as a mean ± standard deviation where data is normally distributed and as a median (interquartile range) for skewed data. Categorical variables are presented as frequencies and percentages. Spearman correlation was used to evaluate possible associations between HbA1c and CFR, change in CBF, or change in CAD. Differences between groups were analyzed using the one-way ANOVA for continuous variables and Chi squared test for proportions. After univariate and age–sex adjustment, we consider variables significant at *p* 0.10 with clinical plausibility to perform a logistic regression model for the prediction of CMD. For that purpose patients were categorized according to the presence/absence of independent CMD (CFR after intracoronary adenosine <2.5) and presence/absence of dependent CMD (% of CBF increase after intracoronary acetylcholine <50 %). We considered shrinkage estimates to assess and avoid over fitting of the model. *p*-values of less than 0.05 were accepted as significant. All statistical analyses were carried out using JMP 9.0 (SAS Institute, Cary, North Carolina, USA).

## Results

**Fig. 1:**
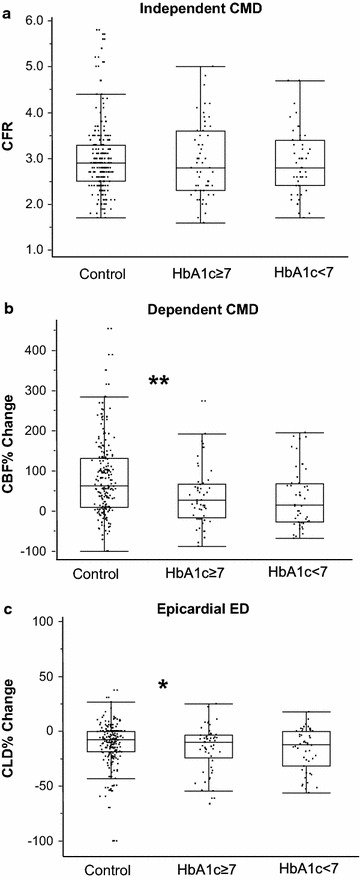
Coronary microvascular and epicardial endothelial function according to HbA1c level. **a** Values of CFR (endothelial independent CMD) according to glycaemic control. **b** Values (in *percentages*) of increase in CBF after Ach intracoronary infusion (endothelial dependent CMD). **c** Values (in *percentages*) of increase in CLD after Ach intracoronary infusion (epicardial endothelial dysfunction). *CFR* coronary flow reserve; *CMD* coronary micro vascular dysfunction; *CFR* *<* *2.5* endothelial independent CMD; *CBF%Ach* percentage of increase in CBF after intracoronary acetylcholine infusion; *CBFAch* *<* *50* *%* endothelial dependent CMD; *CLD%Ach* percentage of increase in coronary lumen diameter after intracoronary acetylcholine infusion; *CLDAch* *<* *20* *%* epicardial endothelial dysfunction. *p < 0.05; **p < 0.001.

The comparative baseline characteristics of the T2DM study group are presented in Table 1. There were significant differences in most of the variables as well as a higher degree of endothelial dependent CMD and epicardial ED in T2DM patients as compared to controls, Fig. 1. The prevalence and endothelial dependence of CMD found in T2DM patients were as follow: 27 % of patients had not any type of CMD, 24 % patients had both independent and dependent CMD, 11 % had only independent CMD and 38 % patients had only dependent CMD.

**Table 1:** T2DM patients basal characteristics

	Control n 214	T2DM n 100	*p* value
Age (year)	49 (41–58)	54 (48–61)	<0.001
BSA (m^2^)	1.82 ± 0.21	2.06 ± 0.25	<0.001
BMI (kg/m^2^)	26.19 ± 5.37	34.32 ± 7.48	<0.001
Female sex (%)	76.9	61.0	<0.01
Creatinine (mg/dl)	0.9 (0.8–1.0)	0.9 (0.8–1.1)	0.34
GFR c–c (ml/min)	86.7 (69.5–107.1)	112.5 (85.8–144.2)	<0.001
GFR MRDR (ml/min)	73.3 (65.4–86.6)	74.1 (62.1–88.9)	0.96
Risk factors and CAD history (%)			
Current smoker	11.2	9.0	0.69
Hypertension	34.2	69.7	<0.001
Hyperlipidemia	46.9	78.9	<0.001
Postmenopausal	35.2	44.4	0.11
CAD family history	60.5	57.0	0.43
Inflammatory markers			
Lpa (mg/dl)	11.0 (7.0–25.0)	13.5 (7–35.7)	0.16
hsCrP (mg/l)	0.4 (0.2–1.1)	1.0 (0.4–3.8)	<0.001
Homocysteine (µmol/l)	7.0 (6.0–9.0)	8.0 (6.0–10.0)	0.15
Coronary epicardial and microvascular function			
CFR	2.9 (2.5–3.3)	2.8 (2.3–3.5)	0.71
CRF < 2.5 (%)	27.2 %	35.0 %	0.18
CBF%Ach	63.3 (9.4–131.4)	25.2 (−20.5 to 68.8)	<0.001
CBFAch < 50 % (%)	41.3 %	62.0 %	<0.001
CLD%Ach	–7.7 (−18.7 to 0.0)	−11.4 (−27.1 to 3.1)	<0.05
CLDAch < 20 % (%)	21.6 %	32.0 %	0.051
CBFbasal	50.2 (34.0–73.5)	52.6 (37.8–72.6)	0.42
LVEDP (mmHG)	13.5 (11.0–23.0)	17.0 (13.0–26.0)	<0.001
Medication (%)			
BBlockers	24.4	41.0	<0.01
CC blockers	36.0	32.0	0.52
Nitrates	32.2	45.0	<0.05
ASA	50.7	64.0	<0.05
ACE/ARB inhibitor	17.8	34.0	<0.01
Lipid lowering	33.8	59.0	<0.001

*BMI* body mass index, *BSA* body surface area, *GFR c*–*c* Cockroft–Gault glomerular filtrate rate, *CAD* coronary artery disease, *Lpa* lipoprotein A, *hsCrP* high sensitive C reactive protein, *CFR* coronary flow reserve, *CMD* coronary microvascular dysfunction, *CFR* < *2.5* independent CMD, *CBF%Ach* percentage of increase in CBF after intracoronary acetylcholine infusion, *CBFAch* < *50* *%* dependent CMD, *CLD%Ach* percentage of increase in coronary lumen diameter after intracoronary acetylcholine infusion, *CLDAch* < *20* *%* epicardial endothelial dysfunction, *LVEDP* left ventricular end-diastolic pressure, *CC blockers* calcium channel blocker, *ASA* acetylsalicylic acid, *ACE/ARB inhibitor* angiotensin-converting enzyme/angiotensin inhibitor.

The baseline characteristics of the T2DM according to their status of glycaemic control are presented in Table 2. Spearman correlation analyses showed the weak negative correlation between HbA1c and Ach induced CBF% change in total subjects including control and DM patients (Table 3). However, after divided subjects into the control and DM group, HbA1c was not significantly associated with any coronary function parameters, either in control group or in DM group. The prevalence of endothelial independent CMD (29.8 vs. 39.6 %, *p* = 0.40) and endothelial dependent CMD (61.7 vs. 62.2 %, *p* > 0.99) were similar in T2DM patients with optimal vs. suboptimal control respectively, Table 2 and Fig. 1. The mean difference between optimal and suboptimal glycaemic controls for CFR was −0.059 [95 % confidence interval (CI) −0.368, 0.250; *p* = 0.70]. The mean difference for CBF% change between the two groups was −3.91 (95 % CI −33.35, 25.52; *p* = 0.792).

**Table 2:** Basal characteristics according to level of HbA1c

	HBA1c > 7 (n 53)	HBA1c < 7 (n 47)	*p* value
Age (year)	55 (49–61)	54 (47–61)	0.45
BSA (m^2^)	2.04 ± 0.25	2.09 ± 0.25	0.40
BMI (kg/m^2^)	33.95 ± 6.59	34.73 ± 8.42	0.63
Female sex (%)	58.5	63,9	0.54
GFR c–c (ml/min)	108.0 (81.4–144.2)	117.7 (89.2–136.5)	0.79
GFR MRDR (ml/min)	75.9 (62.1–92.8)	71.9 (64.3–83.7)	0.23
Risk factors and CAD history (%)			
Current smoker (%)	13.2	4.2	0.16
Hypertension (%)	57.7	82.9	<0.01
Hyperlipidemia (%)	75.0	82.9	0.33
HDL (mg/dl)	45.0 (37.0–54.5)	45.5 (39.0–54.0)	0.83
Triglycerides (mg/dl)	160.0 (98.0–230.0)	118.5 (86.7–159.5)	<0.05
Inflammatory markers			
Lpa (mg/dl)	14.0 (7.0–35.0)	13.0 (7.0–40.0)	0.74
hsCrP (mg/l)	1.0 (0.4–4.0)	1.0 (0.4–3.7)	0.31
Homocysteine (µmol/l)	8.0 (6.0–9.0)	8.0 (6.0–10.0)	0.30
Coronary epicardial and microvascular function			
CFR	2.8 (2.3–3.6)	2.8 (2.4–3.4)	0.55
CRF < 2.5 (%)	39.6	29.8	0.53
CBF%Ach	27.4 (−16.6 to 67.4)	15.2 (−28.2 to 69.1)	0.79
CBFAch < 50 % (%)	62.2	61.7	1.00
CLD%Ach	−10.0 (−24.8 to −3.4)	−12.5 (−31.8 to 0.0)	0.93
CLDAch < 20 % (%)	28.3	36.1	0.52
CBFbasal	52.9 (33.5–70.4)	51.2 (38.0–73.1)	0.45
LVEDP (mmHG)	18.0 (12.2–24.7)	17.0 (15.0–22.0)	0.71
Diabetes status and treatment characterization			
DM duration (months)	60.0 (24.0–100.0)	41.0 (8.0–90.0)	0.22
Fasting glucose (mg/dl)	148.0 (118.0–179.0)	125.0 (103.7–141.0)	<0.01
Insulin (µIU/ml)	14.0 (6.9–20.6)	10.0 (5.1–18.0)	0.47
DM family history (%)	60.4	47.7	0.29
Insulin therapy (%)	33.9	21.2	0.18
Metformin (%)	44.2	44.4	1.00
Metformin only (%)	28.8	35.5	0.66
Insulin releasing (%)	28.8	22.7	0.49
Medication (%)			
BBlockers	35.8	46.8	0.31
CC blockers	28.3	36.1	0.29
Nitrates	33.9	57.4	<0.05
ACE/ARB inhibitor	30.1	38.3	0.53
Lipid lowering	40.1	70.2	<0.05

*BMI* body mass index, *BSA* body surface area, *GFR c*–*c* Cockroft–Gault glomerular filtrate rate, *ACS* acute coronary syndrome, *CAD* coronary artery disease, *Lpa* lipoprotein A, *hsCrP* high sensitive C reactive protein, *CFR* coronary flow reserve, *CMD* coronary microvascular dysfunction, *CFR* < *2.5* independent CMD, *CBF%Ach* percentage of increase in CBF after acetylcholine infusion, *CBFAch* < *50* *%* dependent CMD, *CLD%Ach* percentage of increase in coronary lumen diameter after acetylcholine infusion, *CLDAch* < *20* *%* epicardial endothelial dysfunction, *LVEDP* left ventricular end-diastolic pressure, *CC blockers* calcium channel blocker, *ACE/ARB inhibitor* angiotensin-converting enzyme/angiotensin inhibitor.

**Table 3:** Correlation between HbA1c level and coronary vascular function

Correlation with HbA1c	In total subjects		In control group		In DM group	
	Spearman ρ	*p*	Spearman ρ	*p*	Spearman ρ	*p*
CFR	0.023	0.68	0.046	0.51	0.006	0.96
CBF% change	−0.166	0.003*	0.015	0.83	−0.055	0.59
CLD% change	−0.028	0.62	0.114	0.096	−0.024	0.82

*CLD* coronary lumen diameter, *CBF* coronary blood flow, *CFR* coronary flow reserve, *HbA1c* hemoglobin A1c.

* Statistically significant.

Table 4 shows the age–sex adjusted analysis for the prediction of CMD with those variables significantly associated to CMD in Table 2. In the multivariate analysis (Table 5), age [odds ratio (OR) 1.10; CI 95 % 1.04–1.18; *p* < 0.001] and female gender (OR 3.87; CI 95 % 1.45–11.4; *p* < 0.01) were associated with endothelial independent CMD whereas glomerular filtration rate (OR 0.97; CI 95 % 0.95–0.99; *p* < 0.05) was found to be significantly associated to endothelial dependent CMD.

**Table 4:** Age–sex adjusted analysis for prediction of CMD in T2DM patients

	Independent CMD *OR (CI 95* *%) p value*	Dependent CMD *OR (CI 95* *%) p value*
Age (year)	1.06 (1.01–1.12) *p* 0.01*	0.98 (0.94–1.02) *p* 0.55
Female sex (%)	3.24 (1.24–9.14) *p* 0.01*	1.36 (0.90–1.02) *p* 0.23
BMI (kg/m^2^)	1.06 (0.99–1.48) *p* 0.07	0.96 (0.52–1.24) *p* 0.25
GFR MDRD (ml/min)	1.00 (0.98–1.02) *p* 0.49	0.97 (0.95–0.99) *p* 0.04*
Risk factors		
Hypertension (%)	1.16 (0.43–3.27) *p* 0.75	1.27 (0.52–3.26) *p* 0.59
Triglycerides (mg/dl)	1.00 (0.99–1.00) *p* 0.87	0.99 (0.99–1.00) *p* 0.61
Diabetes control		
HBA1c (%)	1.21 (0.94–1.59) *p* 0.13	0.88 (0.67–1.12) *p* 0.32
HBA1c < 7%	0.60 (0.23–1.46) *p* 0.26	0.99 (0.43–2.24) *p* 0.98
Fasting glucose (mg/dl)	1.00 (0.99–1.01) *p* 0.67	0.99 (0.98–1.00) *p* 0.20
Medication		
Nitrates	1.50 (0.61–3.76) *p* 0.36	0.59 (0.26–1.36) *p* 0.22
Lipid lowering	1.21 (0.48–3.07) *p* 0.68	1.22 (0.53–2.80) *p* 0.63

*CMD* coronary microvascular dysfunction, *BMI* body mass index, *GFR* glomerular filtrate rate, *CFR* coronary flow reserve, *CRF* *<* *2.5* independent CMD, *CBFAch<50* *%* endothelial dependent CMD.

* Statistically significant.

**Table 5:** Multivariate model for prediction of CMD in T2DM patients

	Endothelial independent CMD	Endothelial dependent CMD
Age (year)	1.10 (1.04–1.18) *p* 0.000*	0.98 (0.94–1.03) *p* 0.647
Female sex	3.87 (1.45–11.4) *p* 0.006*	0.71 (0.29–1.70) *p* 0.451
BMI (kg/m^2^)	1.07 (0.99–1.15) *p* 0.054	
GFR MDRD (ml/min)		0.97 (0.95–0.99) *p* 0.038*
HbA1c%	1.24 (0.96–1.64) *p* 0.101	1.13 (0.36**–**2.22) *p* 0.366

*BMI* body mass index, *CMD* coronary microvascular dysfunction, *CFR* coronary flow reserve, *CRF* *<* *2.5* endothelial independent CMD, *CBFAch* *<* *50%* endothelial dependent CMD, *GFR* glomerular filtrate rate, *HTN* hypertension, *HbA1c* *<* *7* *%* optimal glycaemic control.

* Statistically significant.

## Discussion

The current study demonstrates for the first time that glycaemic control is not an independent predictor of a better coronary microvascular function in T2DM patients with chest pain and non-obstructive coronary angiogram. The finding is in line with the reported discrepancy between the optimal control of DM and cardiovascular events [3] and extend some previous reports performed with remarkable differences in study population and methodology. Two small randomized trials evaluating different therapeutic strategies over peripheral ED did not find any relationship between glycaemic control and ED [16, 17]. However CMD was not evaluated in these studies and the current glycaemic target of Hb1C < 7 % was not necessarily achieved [11]. Two studies that addressed the association between HbA1c levels and CFR as evaluated with Doppler echocardiography [7, 18] found no differences between T2DM patients with a good vs. poor glycaemic control, but one reported an improvement of CFR after 6 months in those patients who achieved HbA1c < 7 %. These authors did not evaluate the endothelial dependent CMD and moreover reported differences in study population and methodology may account for the discrepancy. An optimal blood sugar control does not reduce the incidence of epicardial coronary artery spasm in DM patients with chest pain and normal coronary angiogram [8]. Our study extends this previous report to a different population [19] and more importantly, to the coronary microcirculation.

The endothelium independent CFR in response to adenosine did not differ significantly between our subgroup of T2DM patients and the control group (Table 1) as well as between those T2DM patients with optimal vs. suboptimal control (Table 2). The mean values are essentially above the conventional cut-off of normality in a population study of patients with chest pain and non-obstructive coronary angiogram. Since there is significant differences in the CBF increase in response to intracoronary acetylcholine infusion between our subgroup of T2DM patients and the control group, this finding underscore the importance of endothelial function and supports that in similar T2DM patients, endothelial dependent CMD may be the primary mechanism for ischemia. Moreover, according to previous reports, there is conflicting evidence of the predictive value of CFR for cardiovascular events, however the predictive value of epicardial and micro vascular ED in predicting major cardiovascular events is well established [20, 21].

Patients with T2DM and optimal glycaemic control showed a higher prevalence of hypertension. Our study specifically excluded patients with uncontrolled hypertension, left ventricular hypertrophy or renal disease. This fact may possibly explain the lack of association between hypertension and CMD in our study, since the blunting of endothelium-independent CFR and dependent CBF in hypertension is associated with left ventricular hypertrophy [22–25]. Also, those patients were more frequently treated with drugs that have shown to improve endothelial function [26–28].

Our data argue against different prevalence of CMD between T2DM patients with an optimal vs. poor glycaemic control based on the current guidelines recommendations of HbA1c < 7 %. The present study evaluated only the effect of chronic sustained hyperglycemia through a single measurement of HbA1c at the time of catheterization, an accepted reliable marker of the overall exposure integrating fasting, postprandial glycaemic state [29] and mean plasma glucose level [30]. Previous studies have addressed the limited value of the assessment of HbA1c variability [31] while others have emphasized the role of acute glycaemic fluctuations from peaks to nadir as a cause of excessive protein glycation, activations of oxidative stress [32] and ED in DM patients [33]. Our study would be in accordance with this reported evidence supporting that in order to reduce coronary micro vascular complications the target for an optimal glycaemic control should be improved and new ones should be identified [3, 29, 34]. Measures could possibly include reduction of glycaemic variability through self-monitored blood glucose, real time continuous glucose monitoring or the use of new drugs like incretins, with proposed but yet unproven glucose-independent beneficial effects on the endothelium and CV outcomes [35–37]. However, an individualized approach with careful consideration not only to glycaemic control but also to a comprehensive vascular [16] and endothelial health as the final pathway of any possible injury may be necessary to perform successful intervention strategies in T2DM patients. The assessment of ED through peripheral and/or direct coronary measurement in T2DM patients would be therefore a unique tool to obtain that important information about individual patient risk and vulnerability.

## Limitations

The study population comprises T2DM patients with chest pain suspected of cardiac ischemia who were referred for coronary angiography to a tertiary referral center by an independent cardiologist. Therefore the prevalence, severity and reversibility of CMD may be different to other T2DM patients without chest pain. Specifically, our results could not be generalized to diabetes patients with significant CAD, and those with depressed LV function and/or LVH who were excluded in our study. Second, the study was a cross sectional design and does not allow longitudinal study of the effect of glycaemic control on endothelial function. Third, our negative findings may be related to statistical power. However, the confidence intervals for the mean differences in CFR and CBF%Ach between the two groups seem to rule out large differences. Fourth, despite the retrospective nature of our analysis, the collection at the time of catheterization of clinical, laboratory and the direct measurement of CMD was prospectively recorded with a carefully planned protocol that minimized missing and/or error values. The role of new drugs for glycaemic control as well as the effect of a sustained optimal HbA1c level along with glycaemic fluctuations in the long-term is out of the scope of any retrospective analysis and should be clarified in future prospective studies.

## Conclusion

The current study demonstrates that in patients with T2DM with preserved left ventricular function but not coronary artery disease or left ventricular hypertrophy, appropriate glycaemic control does not predict a better microvascular endothelial function. The results may explain the discrepancy between glycaemic control and cardiac events and suggest that vascular function may serve as a therapeutic target in patients with DM.

## Abbreviations

CAD: coronary artery disease
CBF: coronary blood flow
CFR: coronary flow reserve
CI: confidence interval
CMD: coronary micro vascular dysfunction
ED: endothelial dysfunction
HbA1c: hemoglobin A1c
OR: odds ratio
T2DM: type 2 diabetes mellitus
